# Telephone-based group mental health support for older adults in central Canada: pilot acceptability and effectiveness findings of The CONNECT Program

**DOI:** 10.3389/fpubh.2025.1541583

**Published:** 2025-09-05

**Authors:** Kristin Audrey Alison Reynolds, Kira Kudar, Jarod Joshi, Inga Christianson, Dylan Davidson, Georgia Gopinath, Lesley Koven, Corey Mackenzie, Stacey Miller, Nancy Newall

**Affiliations:** ^1^Department of Psychology, University of Manitoba, Winnipeg, MB, Canada; ^2^Department of Psychiatry, University of Manitoba, Winnipeg, MB, Canada; ^3^Department of Clinical Health Psychology, University of Manitoba, Winnipeg, MB, Canada; ^4^A&O: Support Services for Older Adults, Winnipeg, MB, Canada; ^5^Department of Psychology, Brandon University, Brandon, MB, Canada

**Keywords:** older adults, loneliness, mental health, psychological flexibility, group intervention, telehealth, quantitative, qualitative

## Abstract

**Background:**

Though experiences of anxiety, depression, loneliness, and social isolation are prevalent among older adults, treatment access is limited. In central Canada, based on participatory program development methods, our team of researchers, clinical psychologists, and community experts have developed and piloted a novel 6-session telephone-based group intervention called The CONNECT Program, based on the principles of Acceptance and Commitment Therapy (ACT), self-compassion, and psychosocial theories of successful aging.

**Methods:**

We offered The CONNECT Program by telephone from September 2020 to February 2022, completing 7 intervention groups with *N* = 34 participants. We collected quantitative data at baseline and quantitative and qualitative data post-intervention by telephone.

**Findings:**

Overall, our program was deemed to be feasible and acceptable by participants. Pre- to post-CONNECT, we found significant improvements in depression (d = 2.4), emotional support (d = 5.2), mental health literacy (d = 3.2), and psychological flexibility (d = 7.0), indicating large treatment effects. Through conventional content analysis of post-CONNECT individual interviews we developed three main themes: Accessibility (sub-themes: join from the comfort and anonymity of the telephone lines; reduction of age-related barriers), Connectedness (sub-themes: connection to group facilitators; group members; and new information), and Perceived Program Benefits (sub-themes: behavioral changes; emotional and cognitive changes; enhanced mindful awareness).

**Discussion:**

The CONNECT Program is a novel, accessible, and acceptable intervention that demonstrates promise in improving key social and mental health indicators. The findings from this pilot study will inform a future trial comparing The CONNECT Program with standard community programs typically offered to older adults in these provinces. This trial will also evaluate two delivery modes (telephone and videoconferencing) to compare their relative impact and feasibility.

## 1 Introduction

Many countries across the globe are experiencing unprecedented rises in life expectancy, contributing to aging societies (1). One study, published on global life expectancy in *The Lancet* highlights that between 1950 and 2017, life expectancy increased from 48.1 years to 70.5 years for men and from 52.9 to 75.6 for women (2). In Canada, adults ages 65 years and older are expected to comprise ¼ of the population by 2036, growing to almost one third of the population by 2061 (3). However, quality of life, including our connections with others, is as or more important when compared to quantity of years lived.

Population-based research from Canada and the United States indicates that between 3 and 7% of adults ages 55 years and older meet diagnostic criteria for a past-year mood disorder, and between 3 and 11% of older adults meet criteria for a past-year anxiety disorder (4, 5). Prevalence rates of subsyndromal mental health problems in which symptoms are elevated but diagnostic criteria are not met in full, are much higher among older adults, and impact negatively on quality of life (6, 7). For example, subsyndromal depression is approximately 2–3 times higher than clinical depression (major depressive disorder) among older adults (8). Late-life mental health problems can be severe, with men ages 65 years and older having the highest rate of completed suicide in Canada (9).

Though individuals may experience mental health problems such as anxiety and mood disorders in later life for a variety of reasons, social isolation and loneliness are associated with the development and persistence of such problems (10–12). Social isolation refers to having few or no social interactions and lower quality of relationships (13), and loneliness refers to perceived social isolation (13). Pre-COVID-19, approximately 20% of adults ages 65 + were isolated; 10% were lonely often or always; and 8% could be considered very or extremely isolated (14, 15). With COVID-19 restrictions to older adults’ in-person contact and activities, social isolation and loneliness have only increased (16–19), accompanied by worsened rates of depression and anxiety (20).

Importantly, although older adults experience high rates of mental health problems, loneliness, and isolation, they are less likely to seek professional psychological help compared to younger age groups (21, 22), with approximately 70% with prevalent anxiety and mood disorders not using any services (23). Further, research from our group has shown that when older adults do seek psychological help, they may experience challenging routes to care, ‘muddling through’ from one treatment option or service to the next (24, 25). Though there are many complex barriers to seeking treatment for mental health problems, one important finding demonstrated consistently in the extant literature is that older adults have lower rates of mental health literacy compared to younger age groups—meaning that they are less likely to accurately label symptoms of a mental health problem, and they identify fewer sources of treatment for mental health problems. Survey research by our group, with a sample of approximately 250 older adults from central Canada highlighted that over 57% of our sample reported being unfamiliar with the types of treatment for mental health problems (26). This finding reinforces the importance of programs aimed to improve mental health literacy among older adults and those close to them, as well as the need for programs within the community to target mental health—as older adults may be more likely to seek and receive community-based support as compared to formal mental health services (27, 28).

Social participation through community-based activities has the potential to increase social engagement and enhance mental health among older adults [e.g., (27, 29–31)]. Participation in activities in one’s social environment and higher frequency of social contact is associated with lower rates of depression, generalized anxiety disorder, and cognitive impairment (32). Recent research by our group, using nationally representative data from the Canadian Longitudinal Study on Aging (CLSA; *N* = 51,338 ages 45–85) found that in comparison to those who endorsed infrequent/no participation in community programming, more frequent participation was associated with greater social support, higher cognitive abilities, increased satisfaction with life, fewer depressive symptoms, reduced odds of self-reported mood and anxiety disorders, and fewer self-reported physical conditions (26). Importantly, participation in community-based activities and programs has been greatly impacted by the COVID-19 pandemic [e.g., (33–35)]. A recent data report by Statistics Canada noted that the mental health impacts of physical distancing associated with COVID-19 may be experienced most prominently by older adults who are marginalized, including those with lower socioeconomic status and those with disabilities and mobility issues, making accessibility to community services particularly important among these segments of the population (36).

Barriers to participating in community programming pre-dated the COVID-19 pandemic, as most community programs were offered in-person, posing a significant barrier for older adults who experience challenges with participating in activities outside of their homes (37–42). There is a growing focus on technology-based community interventions, including those offered by telephone, Internet, and iPad/smartphone applications, with findings demonstrating promising results in reducing social isolation among older adults (43–45). Telephone-based interventions may offer similar advantages to online interventions, while also reaching a broader audience. In one study, researchers evaluated a not-for-profit telephone service where volunteers engaged in casual telephone conversations with isolated older adults. Participants reported increased confidence, community engagement, and social activity (46). Further, research by our group suggests that frequent participation in telephone-based community programming for older adults is effective in reducing social isolation (47).

One telephone-based program available in Manitoba, Senior Center Without Walls (SCWW), has been offered by A & O: Support Services for Older Adults (A & O) since 2009. Based on a similar program developed in San Francisco, it was the first of its kind in Canada. The aim of SCWW is to improve the reach of community programming through telephone, which older adults can access from their homes. The program is offered to those residing in a central Canadian province who are 55 years of age and older and provides access to a variety of activities (e.g., sing-alongs, games, educational lectures) over the telephone. SCWW activities are facilitated by individuals from various backgrounds, including musicians, artists, health professionals, and A & O staff and volunteers. In 2011, researchers from our group sought to examine, via qualitative telephone-based interviews, the characteristics of those participating in the SCWW program and evaluate whether the program was meeting the intended aims of reaching those who were experiencing social isolation or loneliness. Findings indicated that the program was reaching its target audience, and participants described feeling enhanced connections to community and increased knowledge about the various topics discussed during the sessions. Importantly, participants expressed a desire for more programming focused on mental health (47).

## 2 Objectives

This research seeks to evaluate the feasibility, acceptability, and initial outcomes of a pilot offering of The CONNECT Program in a central Canadian province.

### 2.1 Ethical considerations

Ethics approval was obtained from the central Canadian university in which this study took place. The participants provided their verbal consent to participate in this study.

### 2.2 Materials and methods

Our group of clinical psychologists, expert researchers in health and aging, and our community-based partner developed a telephone-based group mental health program for older adults experiencing loneliness, social isolation, and co-occurring mental health problems (i.e., anxiety and depression). We followed best practice approaches in participatory program development methodology in creating the program, including hearing from multiple stakeholder groups, having open-ended discussions, meeting several times with each stakeholder group to ensure voices are heard, and reviewing visuals such as a program logic model. We held focus groups with three key stakeholder groups: (1) adults ages 65 + experiencing challenges with loneliness, social isolation, low mood, and anxiety symptoms; (2) staff and volunteers working with A & O: Support Services for Older Adults; and (3) mental health professionals working with older adults (i.e., geriatric mental health clinicians, psychologists, psychiatrists, social workers). We heard from these three groups about the issues faced by older adults, their mental health-related needs, supports available (and lack thereof), and interventions that could be developed to meet their needs. Based on focus group findings, we developed a program logic model, which included possible Inputs/Resources (i.e., telephone program platform, program facilitator/clinical psychologist, group material, research assistants); Activities (i.e., setting ground rules, introductions, mental health literacy, understanding challenging thinking patterns, setting goals, learning behavioral responses to managing anxiety and low mood, and exploring helpful resources in one’s community); Outputs (i.e., session attendance, session homework completion); and Outcomes (i.e., reducing anxiety, depression, loneliness, isolation). This logic model was reviewed in a second round of focus groups with each of the stakeholder groups. Input from the stakeholder groups on the program logic model was categorized into a thematic framework, and included the main themes of: Need for social engagement; Need for accessible mental health community programming; Program components (i.e., psychoeducation on mental health problems, loneliness, and social isolation; developing coping strategies; formation of life- and health-related goals); and Program processes (i.e., socializing and community building; alliance-building; and fostering a safe environment). These discussions and themes led to our co-development of a telephone-based group intervention called The CONNECT Program:

**C**reating.

**O**pportunities to build social.

**N**etworks, learn.

**N**ew skills to manage challenging emotions.

**E**nhance mindful awareness and acceptance of emotions, & increase self-.

**C**ompassion, through.

**T**elephone-based group programming.

Integrating Acceptance and Commitment Therapy (ACT) (48–51), self-compassion (52), and psychosocial theories of successful aging, we created a client workbook and facilitator manual for a 6-session telephone, group-based program for older adults experiencing loneliness, social isolation, and co-occurring mental health symptoms (i.e., anxiety, depression). We followed Hays’ tri-flex model of psychological flexibility, which includes three core aims and skills of: *Open Up*, *Be Present*, and *Do What Matters*. ACT is among the most empirically supportive cognitive behavioral therapies, with mounting evidence supporting its effectiveness for a range of challenges involving psychosocial impacts (53). Each session took place over Webex audio, was 90- min long, and included experiential activities, with a new mindfulness activity each week, group discussion, presentation of a new skill, summary of information, and a skill to practice over the week prior to the next session (see Table 1). In line with best-practice methods in group therapy facilitation, we included between 5 and 8 participants in each group, two to three group facilitators, including one Clinical Psychologist and two clinical psychology graduate students, and allowed for the presence of important group process factors to occur [i.e., installation of hope, universality, imparting information, group cohesion; (54)].

**Table 1:** Overview of session structure and activities in The CONNECT Program.

Session #	Title	Sample activities
1	Introduction to The CONNECT Program	Welcome, Notice 5 Things, Mental health & loneliness, Session structure
2	Connecting with the Present Moment & with Yourself by Opening Up to Challenging Emotions	Mindful coffee break, What do you miss out on by not being present, What does it mean to be present, The struggle with loneliness
3	Defining Values: What’s Guiding You?	Passengers on a Bus, Review of willingness, What are values, Values Crokinole Board
4	Watching your Thinking and Practicing Self-Compassion	Lemons Lemons Lemons, Getting unhooked, Practicing self-compassion
5	Taking Action by Setting Goals: Select, Optimize, Compensate	Leaves on a Stream, Goal setting, Willingness & Action Plans
6	Connecting with Others and CONNECT Summary	Challenges to connection, CONNECT solutions, Resources, Next steps

There is an absence of accessible and evidence-based group mental health programs offered by telephone to meet the needs of lonely and socially isolated older adults who are also experiencing challenges related to their mental health. The CONNECT Program, grounded in the needs of adults ages 65 + with lived experiences of social isolation, loneliness, and co-occurring mental health symptoms, as well as the perspectives of important stakeholder groups, was created to fill these identified gaps in research and practice.

### 2.3 Methods

Inclusion criteria included: adults ages 65 + residing in a central Canadian province, who identified minimal threshold level of social isolation (55, 56), loneliness (57), anxiety (58, 59), and depression (58, 59) through telephone-based screening. Exclusion criteria included: age younger than 65, residing outside of the central Canadian province, self-reported major or minor neurocognitive disorder and other forms of serious mental illness, and not being able to provide consent independently. Adults ages 65 + were recruited through notifications placed in the newsletters of community-based organizations (e.g., active living centers, senior centers). Interested participants called the study research coordinator and were screened for eligibility in terms of the criteria indicated above. If participants were deemed eligible, the research coordinator reviewed the consent form with them over the telephone and sent a copy to them via email or mail depending on their preference.

We employed a convergent parallel mixed methods design, where quantitative and qualitative data were collected and analyzed separately, followed by a comparison and integration of findings examining unique and overlapping results. This approach was taken to maximize our understanding of participants’ experiences in The CONNECT Program (60). All questionnaire and qualitative interview data were collected via telephone by the research coordinator or research assistant. Between 1 and 7 days prior to participation in their first CONNECT Program session, participants were contacted by the study coordinator to complete a baseline questionnaire. Directly following the final group session (6th session), participants completed a post-CONNECT questionnaire (measures described below). Participants were also invited to complete an individual qualitative exit interview, which ranged from 60 to 90 min in length. Participants who completed the baseline questionnaire prior to the CONNECT group received a $10 gift card, and participants who completed the post-CONNECT questionnaire received an additional $10 gift card. Participants who completed the qualitative interview post-CONNECT received an additional $10 gift card.

#### 2.3.1 Measures

The baseline questionnaire included demographic questions (i.e., age, gender identification, highest level of completed education, occupational status, marital status, and racial or ethnic background), the three-item loneliness scale (57), the PROMIS social isolation 8a (55), PROMIS emotional support (61), PROMIS anxiety short form 4a (58, 59), PROMIS depression short form 4a (58, 59), the brief measure of mental health literacy (62), and the Acceptance and Commitment Scale-measure of psychological flexibility (63, 64). Reliability analyses were completed with our sample for each of the outcome measures, with all internal consistency Cronbach’s Alpha values in the acceptable to excellent range (i.e., loneliness = 0.89; social isolation = 0.85; emotional support = 0.89; anxiety = 0.87; depression = 0.89; mental health literacy = 0.87; and psychological flexibility = 0.92). The post-CONNECT questionnaire included these measures, with the addition of questions concerning program feasibility and acceptability. The interviews included open-ended questions to better understand participant experiences in The CONNECT Program. Interview questions included: Tell me about your participation in CONNECT; in what ways did your participation map on (or not) to your needs; what aspects did you like and dislike about this program being delivered by phone; describe your experiences interacting with the other group members; describe your experiences interacting with the program facilitators; how did you experience the program content; what impact has the program had on you (if any); and do you have any suggestions that would be helpful in improving the program. Responses were probed when brief, and were elaborated upon by asking, “can you tell me more about that” and “can you provide an example of that.”

#### 2.3.2 Analytic approach

IBM SPSS for Mac (Version 29.0.2.0) was used to organize and analyze quantitative data. Participants responses for quantitative survey data was summarized using descriptive statistics. T-tests and effect sizes (Cohen’s d) were completed to analyze participants’ pre-post CONNECT outcomes. Qualitative interviews were audio-recorded, transcribed with the assistance of Trint Software, and analyzed using conventional content analysis, an inductive qualitative approach (65). As the main purpose of the qualitative interviews was to gather more information to understand participant experiences and help to inform program revisions, it was important for our team to select an analytic approach that allowed us to stay grounded to the data, developing themes based on the interviews as opposed to pre-existing theories or frameworks. Conventional content analysis allowed for a rigorous coding process generating clear key themes and sub themes. The first author, in addition to two research assistants, participated in qualitative analysis. Analysts reviewed transcripts independently and met regularly to explore their various interpretations of the data, collaborating to strengthen their understanding of participant experiences of The CONNECT Program. The coding process was applied in a fluid, iterative method, and included: Familiarization (listening to the audio-recordings of interviews, reading interview field-notes, reading through the transcript several times and jotting down analytic thoughts); Generating initial codes (combing through the transcripts staying close to the data while documenting initial codes); Generating themes (listing initial codes and discussing, as a coding team, overlap and possible thematic categories); Reviewing themes (examining drafted thematic categories and refining categories with subsequent analysis of incoming interviews, while going back to add nuance to the main and sub-themes); and Defining and naming themes (finalizing the names of themes, including definitions and quotations). Once our thematic framework was developed, we entered main themes and sub-themes into NVivo 14 for Mac and re-coded qualitative data to further organize participant quotes and to ensure that each thematic element was thoroughly represented with our data. Qualitative rigor was enhanced due to the analytic method followed, documentation of coding notes (audit trail), and involvement of several lenses and perspectives in the final development of the thematic framework.

## 3 Results

As part of our pilot research, we offered The CONNECT Program by telephone from September 2020 to February 2022, completing 7 intervention groups with a total of N = 34 participants with an average age of 72 (age range of 60–93), including 32 woman-identifying participants and 2 male-identifying participants. Please see Table 2 for a complete description of participant sample characteristics. Overall, the program was deemed to be feasible and acceptable by participants. For example, participants rated that they were satisfied with facilitator expertise (91%), time of day the program was offered (88%), and the process of receiving assessments over the phone (79.4%). Lowest-rated items of satisfaction included number of sessions (62% satisfied) and interaction with other group members (47% satisfied), with participants noting a desire for more sessions and more interaction. Across all treatment groups, we had two participants end their participation in The CONNECT Program prior to the last session, one due to hospitalization for a physical health challenge and another noting that they were not able to make the time commitment at this time. Please see Table 3 for further description of feasibility and acceptability as rated by participants. Regarding initial program effectiveness, from pre- to post-participation in The CONNECT Program, we found significant improvement in depression (t (32) = 2.95, *p* < 0.01, Cohen’s d = 2.4); emotional support (t (33) = 2.06, *p* < 0.05, d = 5.2); mental health literacy (i.e., knowledge concerning the recognition, prevention, and management of mental health problems; t (31) = 3.06, *p* < 0.05, d = 3.2), and psychological flexibility (t (32) = 2.45, *p* < 0.01, d = 7.0). Please see Table 4 for identification of means and standard deviations of these outcome variables.

**Table 2:** Sample characteristics of *N* = 34 CONNECT program participants.

Age, M (SD)	72.1 (8.1)
Gender Identification, N (%)	
Female	32 (94.1)
Male	2 (5.9)
Highest Level of Education, N (%)	
Grade 12 or less	12 (35.3)
College/trade school	12 (35.3)
University undergraduate degree	7 (20.6)
University graduate degree	3 (8.8)
Marital Status, N (%)	
Single/Widowed	19 (55.9)
Married	9 (26.5)
Divorced	6 (17.6)
Living Arrangement, N (%)	
Living alone	22 (64.7)
Living with partner	7 (20.6)
Living with adult child	5 (14.7)
Location of Residence, N (%)	
Urban	30 (88.2)
Rural	4 (11.8)
Ethnic and cultural origins, N (%)	
Canadian	24 (70.6)
French-Canadian	3 (8.8)
Indigenous	2 (5.9)
Asian	2 (5.9)
Black or South Asian	2 (5.9)

**Table 3:** Participant-rated feasibility and acceptability (*N* = 34).

Scale item	(% Satisfied)
Steps performed to reassure privacy and confidentiality	32 (94.1)
Expertise of the facilitators	31 (91.2)
Time of day at which program was offered	30 (88.2)
Content and theme of sessions	29 (85.3)
Number of participants in the group	29 (85.3)
Process of receiving assessments over-the-phone	27 (79.4)
Mindfulness exercise at the beginning of each session	25 (73.5)
Homework and take-home exercises	25 (73.5)
Time allocated to group discussion	24 (70.6)
Length of sessions	22 (64.7)
Number of sessions	21 (61.8)
Interaction with other group members	16 (47.1)

**Table 4:** Participant-rated effectiveness indicators (*N* = 34).

Outcome variable	Pre-CONNECT M (SD)	Post-CONNECT M (SD)
Acceptance and Action (AAQ)*	25.7 (9.8)	22.5 (7.4)
Mental Health Literacy	11.5 (3.2)	13.5 (3.5)
Anxiety	10.6 (3.5)	9.7 (3.7)
Depression	9.9 (3.9)	8.6 (3.3)
Loneliness	6.8 (1.9)	6.5 (1.9)
Social Isolation	22.5 (6.4)	21.7 (6.6)

*AAQ: Higher scores indicate greater experiential avoidance and lower psychological flexibility.

With the goal of understanding program feasibility, acceptability, and outcomes from the unique voices of participants, we completed in-depth, semi-structured qualitative interviews via telephone with 20 participants. Content analysis of qualitative data led to the development of three main themes, converging to understand what allowed participants to join and stay involved in The CONNECT Program: Accessibility (sub-themes: join from the comfort and anonymity of the telephone lines; reduction of age-related barriers), Connectedness (sub-theme: connection to group facilitators, group members, and new information), and Perceived Program Benefits (sub-themes: behavioral changes; emotional and cognitive changes; enhanced mindful awareness).

*Accessibility*. Participants shared how the program’s telephone-based.

delivery helped to increase access to group programming and enhanced their overall program engagement. Participants expressed that receiving the program over the telephone allowed them to take part. Participants also described increased feelings of openness due to perceived anonymity of the telephone. Participants described a reduction of age-related barriers to service use, particularly as they related to those living in central Canada where the climate is extreme (cold winters and hot summers), and individuals may be living in remote communities where travel is difficult. One participant described:

“It’s very convenient, meaning that it’s low tech and then convenient in another way where I do not have to travel. I do not have to go anywhere. Very convenient. And it’s nice if you are shut in. And the big advantage is there’s no excuses. So you get a phone call. Well, you know, if you made a commitment, you there, you are basically there. So you know, there’s those are the positives.” (P20).

In line with this quote, one participant who lived rurally noted:

“I’m not in a city, so everything means driving to the city, right. At any meeting or and I’m not driving anymore. So it’s having to depend on someone to drive me, have someone available to drive me. So I think it’s a big reason that it was accessible.” (P4).

Describing their experience of increased accessibility, increased comfort and safety, and reduced age-related barriers, another participant voiced:

“Mobility can certainly be a barrier and distance is another one. The phone, you know, it’s a toll-free line. You do not have to go anywhere. You can be comfortable in whatever setting you choose at home, whether you are sitting standing in line. You know, again, there’s no judgment. You are at the group, and it is what it is. So, there’s, again, more safety.” (P2).

Further describing the importance of anonymity to them, another participant highlighted:

“I’m more comfortable with that anonymity of a telephone because all they are hearing is my voice. Maybe some of the things that I say, but it’s not as if they are seeing me and judging me by my appearance or who’s around me. It’s all about the voice and what I’m expressing.” (P1).

As a final example highlighting and unifying the importance of joining from the comfort and anonymity of the telephone lines and reduction of age-related barriers.

It’s more informal [having the program delivered over-the-phone], I mean, you can be in your pajamas, you could be eating a piece of cheese … You do not have to get a ride to go there. It’s just you answering your phone and that’s it.” (P5).

*Connectedness*. Participants described their experience of connection to group facilitators, group members, and to new learnings throughout their involvement in The CONNECT Program. When describing their participation, P4 described:

“I like the fact that we were connecting. I was connecting with people…it was an inclusive group, male and female. … I enjoyed the fact that we were connected.”

Another participant noted that the joined the program with the aim of connecting with others. She voiced:

“In this program - as much as we are not, we are not physically together. That’s another voice at the end of the phone that I can talk to that I could interact with. So I did it to get more human contact. And I thought that the space program was extremely good for me.” (P12).

One participant described an overall positive experience interacting with and learning from the group facilitators and members:

“I know I’ve gotten a lot of information from other people [group member] and you people [facilitators], I’ve learned how to do things that I normally would not do. And I’ve learned a great deal from this program.” (P2).

Conversely, several participants described their desire for more connection with group members, and more time spent on open discussion and relationship building. For example, one participant expressed, “It’s called CONNECT, and there wasn’t enough connection.” (P1).

Another participant shared that they enjoyed the interactions between group members throughout the program, however, wanted them to talk more:

“I wanted them [the group] to talk more. They would not share, or they could be the type or, you know, that it was ingrained in them not to share, like keep the stiff upper lip kind of mentality.” (P4).

In describing this search for connection and the challenge of The CONNECT Program in allowing them to realize this goal, one participant described:

“I really would have preferred more interaction. I do not know how it could have been done. I was hoping to get some sort of connection with other people. That’s what I was looking for.” (P15).

*Perceived Program Benefits*. Participants discussed behavioral changes, emotional and cognitive changes, and enhanced mindful awareness as ways in which they saw themselves grow throughout participation. One participant described changes in their behaviors in line with program material when stating:

“It’s reinforced my belief in physical activity and being more active, being more making more phone calls. You know, these are the things that we’ll have to keep up. I just I’ve just, you know, like what are the things that I can do to make my life better.” (P20).

Another participant voiced, “For the first time in my life I actually tried to pinpoint down what are my values, and that’s yeah, because I was never really clear to me. So I went online and got a whole bunch of ideas. But that kind of has started a new search for me. Like, are these really my values for me? So it’s led to a bit of self-understanding.” (P11).

Another participant highlighted:

“That particular exercise [values-based crokinole board exercise] gave me the jumpstart to get motivated to get because I was in a slump, you know, like to say home caring. Losing my energy, you know, feeling down. So that crokinole board exercise gave me the starting point to get busy and start reaching out again, not just sitting here. I’m out of my slump and I’m doing things again. So I found that they kind of give me the shot in the arm. Like, and not to take things seriously, like leaf it’s floating down the river, goodbye, you know, there will be more leaves, you know, in time to come again that will float and go away.” (P20).

Some participants shared how their involvement in the program has changed their way of thinking, affective state, and personal mindset. Participants voiced difficulty with negative thinking and reluctance to acknowledge or express their feelings (with themselves and with others). For example, one participant described:

“At the time that I heard about the program, I felt like I was kind of going down a slippery slope that would lead to clinical depression because I’ve been there before. I found that I wasn’t connecting with people. I wasn’t reaching out to people. I wasn’t doing a lot of things I used to enjoy. And when I heard about this program, I thought that it would help me. And it has very much. I’ve learned a lot about myself and my thought process. And I think it has done me a lot of good.” (P2).

In line with this experience, another participant voiced the impact of the material and program to their self-learning:

“I tend to beat up on myself quite a bit. I have a lot of negativity in my life, so that was pretty important. I think that was the biggest one.” (P18).

Enhanced mindful awareness was a third way in which participants described experiencing benefits or growth from involvement in The CONNECT Program. One participant noted,

“Sitting comfortably and thinking about your surroundings in that every time outside with the dogs, I remember that. Because that was something that. That really kind of hit home with me with this mindfulness exercise. And I’ll probably continue doing that for the rest of my life because it’s just so good.” (P12).

Another participant shared their beneficial experience with mindfulness exercises when voicing:

“The one with my senses, you know, you just get in the moment and with, you know, with my senses what I could see. You know how cool it was if there was a breeze, I had the window open or, you know, the smell, like if I’m sitting, I’m in my bed and I’m feeling the sheets and, you know, like, I’m comfortable. It helped me to get in touch with how I felt. And then when I touch a cup of coffee now, it’s not the same either, because, you know, so I have that exercise and the river and the banks and yeah, I loved that one too. So yeah, I loved those mindfulness exercises.” (P16).

Most participants expressed a need for more time, suggesting that in the future, facilitators should either increase the length of each session or add more sessions to the program’s current curriculum:

“Extend it [the CONNECT program] for a couple of weeks as well. What is really a couple of hours more when you think about it and it’s still connecting with people. And yeah, more discussion and draw the questions, those thought-provoking questions that would make people think and draw stuff out.” (P2)

## 4 Discussion

Quantitative and qualitative findings, individually and convergently, provide preliminary evidence for the feasibility, acceptability, and positive outcomes associated with CONNECT Program involvement for adults ages 65+. Quantitative findings highlight feasibility in initiating and maintaining involvement in The CONNECT Program, joining telephone-based sessions, receiving telephone-based assessments, and connecting with group facilitators and with the content shared in sessions. Preliminary quantitative outcomes as assessed in pre-post outcome measurement include significant differences with large treatment effects in depression, emotional support, mental health, literacy, and psychological flexibility. Qualitative findings support and extend these quantitative findings, by demonstrating, through the voices of participants, why the program was accessible, connective, and beneficial. Quantitative data indicating that the majority of participants were not satisfied with the number of sessions or the connection between group members was supported and elaborated on in qualitative interviews, promoting helpful recommendations to program refinement. Non-significant quantitative results regarding the impact of program participation on anxiety, social isolation, and loneliness, are not in line with qualitative data, which promotes understanding of participant growth in these areas.

The CONNECT Program stands out for its telehealth phone-based delivery, grounded in the Acceptance and Commitment Therapy (ACT) framework, psychosocial theories of successful aging, and principles of group therapy alliance. Specifically designed to meet the needs of older adults experiencing loneliness, social isolation, anxiety, and depression, the program offers a holistic and evidence-informed approach to mental health support. While other studies in this area have contributed important insights, many adopt narrower focuses. For example, some provide telehealth-delivered Cognitive Behavioral Therapy (CBT) or ACT-based treatments tailored to older adults [e.g., (66–68)], however, these often target specific mental health concerns, such as anxiety or depression, in isolation, and for some programs research has mostly explored their feasibility and acceptability rather than their effectiveness. Others emphasize interventions delivered through phone calls or videoconferencing, often focusing on peer support or empathy-based approaches without consistently employing structured, evidence-based frameworks [e.g., (69–71)]. Although studies with more focused approaches may provide valuable depth in exploring specific intervention components, the collective body of research represents a significant step toward advancing mental health interventions for older adults. This growing evidence base highlights the potential for more accessible and effective program delivery, particularly by addressing barriers and identifying enabling factors that enhance intervention outcomes (72, 73).

Our findings are consistent with previous qualitative research on the accessibility and feasibility of telephone programs for socially isolated older adults. For example, the theme of connectedness in our study aligns with sense of belonging (46) and feeling more connected (47). Similarly, perceived program benefits in our research correspond to themes such as impact on mental well-being (47) and alleviates loneliness and anxiety (46). The theme of new information reflects increases knowledge (47), and accessibility is consistent with access/barriers (47). In addition, our study offers unique contributions, this includes practical insights into enhancing accessibility, such as providing comfort and anonymity through telephone lines and reducing age-related barriers, a deeper understanding of connectedness through group interactions and information sharing, evidence of behavioral changes and improved mindfulness, and the educational value of telephone programs for older adults.

As a result of the pilot study, The CONNECT Program demonstrated promising outcomes. Indicators of depression, social support, emotional support, mental health literacy and psychological flexibility showed improvement in the post-program period. The large Cohen’s effect sizes indicate high clinical significance of the program. From pre- to post-participation in The CONNECT Program, we found significant improvement in depression, which aligns with other studies that report reductions in depression [e.g., (66, 69)] as well as improvement in emotional support (69). The improvement in mental health literacy and psychological flexibility, however, has not been reported in previous studies prior to ours.

At the same time, in this pilot study, we did not find significant results for loneliness, social isolation, and anxiety before and after the intervention. This may suggest that the sample size was relatively small, or that the limited program duration and variability in participant needs played a role in these findings.

While some studies explore various technological strategies for reducing social isolation and the associated mental health conditions in older adults, the majority of implemented strategies are internet-based and digital [e.g., (74)]. In contrast, telephone and videoconferencing may be simpler, more accessible, and easier to understand for older adults. These methods can expand the reach of participants, particularly in rural areas, as such interventions are low-cost and low-risk technologies that do not require complex devices or internet access, making them easier for older adults to adopt (75). However, we recognize that telephone-based interventions lack visual components, despite enabling audio communication. So, incorporating video conferencing could potentially enhance the delivery and outcomes of The CONNECT Program.

### 4.1 Limitations

This is a pilot study, so several limitations need to be considered. Due to the absence of randomization and a control condition, we cannot confirm that results are directly attributed to CONNECT program participation, and further trial-based research is needed. The sample size though consistent with published pilot evaluation research was small and homogeneous, with participants exclusively recruited from central Canada. To ensure the generalizability of the results and assess the program’s effectiveness, it would be beneficial to not only increase the number of participants but also diversify our sample according to socioeconomic indicators, gender, cultural background, and geographic variation (e.g., urban, rural). Moreover, only one delivery mode was used—telephone (audio-only)—which may have influenced participants’ experiences and outcomes.

### 4.2 Future directions

The CONNECT Program is planned for implementation across Canadian provinces to evaluate the program’s effectiveness in improving key social and mental health outcomes. Furthermore, the findings from the pilot study will guide the design of a future trial aimed at comparing the effectiveness of The CONNECT Program with standard community programs typically offered to older adults in their respective provinces. The trial will also evaluate and compare two delivery modes - telephone and videoconferencing, to determine their relative impact and feasibility.

Engaging knowledge users, older adults, and their representatives will be a vital step in the future study of The CONNECT Program. Their input will offer insights into their lived experiences and perspectives, which also can guide efforts to strengthen collaboration among patients, researchers, healthcare providers, and decision-makers.

## Data Availability

The raw data supporting the conclusions of this article will be made available by the authors, without undue reservation.
